# Sex-specific differences of humoral immunity and transcriptome diversification in older adults vaccinated with inactivated quadrivalent influenza vaccines

**DOI:** 10.18632/aging.202733

**Published:** 2021-03-19

**Authors:** Jing Yang, Xiaoyuan Huang, Jiayou Zhang, Renfeng Fan, Wei Zhao, Tian Han, Kai Duan, Xinguo Li, Peiyu Zeng, Jinglong Deng, Jikai Zhang, Xiaoming Yang

**Affiliations:** 1 National Institute of Engineering Technology Research in Combination Vaccine, Wuhan 430207, Hubei Province, China; 2 Wuhan Institute of Biological Products Co., Ltd., Wuhan 430207, Hubei Province, China; 3 China Biotechnology Co., Ltd., Peking 100029, China; 4 Guangdong Province Institute of Biological Products and Materia Medica, Guangzhou 510440, Guangdong Province, China; 5 Gaozhou Center for Disease Control and Prevention, Maoming 525200, Guangdong Province, China

**Keywords:** QIVs (quadrivalent inactivated influenza vaccines), sex dimorphism, HAI, DEGs (differential expressed genes), transcripts

## Abstract

Clinical data showed sex variability in the immune response to influenza vaccination, this study aimed to investigate differentially expressed genes (DEGs) that contribute to sex-bias immunity to quadrivalent inactivated influenza vaccines (QIVs) in the elderly. 60 healthy adults aged 60-80 yrs were vaccinated with QIVs, and gene expression was analyzed before and after vaccination. The humoral immunity was analyzed by HAI assay, and the correlation of gene expression patterns of two sex groups with humoral immunity was analyzed. The DEGs involved in type I interferon signaling pathway and complement activation of classical pathway were upregulated within 3 days in females. At Day 28, the immune response showed a male-bias pattern associated with the regulation of protein processing and complement activation of classical pathway. A list of DEGs associated with variant responses to influenza vaccination between females and males were identified by biology-driven clustering. Old females have a greater immune response to QIVs but a rapid antibody decline, while old males have the advantages to sustain a durable response. In addition, we identified genes that may contribute to the sex variations toward influenza vaccination in the aged. Our findings highlight the importance of developing personalized seasonal influenza vaccines.

## INTRODUCTION

Influenza virus is a long-standing global health threat that attacks all countries. Previous studies estimated that 291,243 to 645,832 seasonal influenza associated respiratory deaths (4.0 to 8.8 per 100,000 individuals) would happen annually [1, 2]. Of these, over 60% are older adults [2]. Immunosenescence is thought to reduce the capacity of immune response to pathogens and vaccines, consistent with humoral immunity outcomes after influenza A/H1N1 vaccination in the elderly [3]. Moreover, sex-based different immune responses to virus pathogens and viral component vaccines have been recorded [4–10]. These results suggest inter-individual differences in immunogenicity of seasonal influenza vaccines across populations and emphasize the need of exploiting personalized seasonal influenza vaccine [8, 11].

Immunization outcomes of both humoral and cell-mediated immunity are superior in females than in males for influenza vaccination [6, 12, 13]. Sex-based differences with higher humoral antibody titers accompanied by stronger inflammatory responses to influenza vaccination have been shown in females [5, 14]. However, most vaccine clinical trials did not analyze immune response outcomes based on sex [15]. Sexual dimorphism in immune system has not been analyzed for antibody immune response to inactivated quadrivalent influenza vaccines (QIVs).

One epidemiological study in United States from 1997 to 2007 demonstrated higher risk of influenza associated mortality in males compared to females [16]. Furthermore, the increased expression levels of androgens and genes involved in lipid metabolism resulted in lower humoral immune responses to influenza vaccination in males [17]. Therefore, the analysis of differentially expressed genes before and after immunization will be important to reveal underlying genetic basis of sex dimorphic immune responses to QIVs inoculation in older adults. In this study, we aimed to investigate sex differences in response to inactivated QIVs across the population aged 60 yrs and older, and explore molecular mechanism underlying differential humoral responses to QIVs.

## RESULTS

### Different humoral immune responses to QIVs in old adults

**Figure 1:**
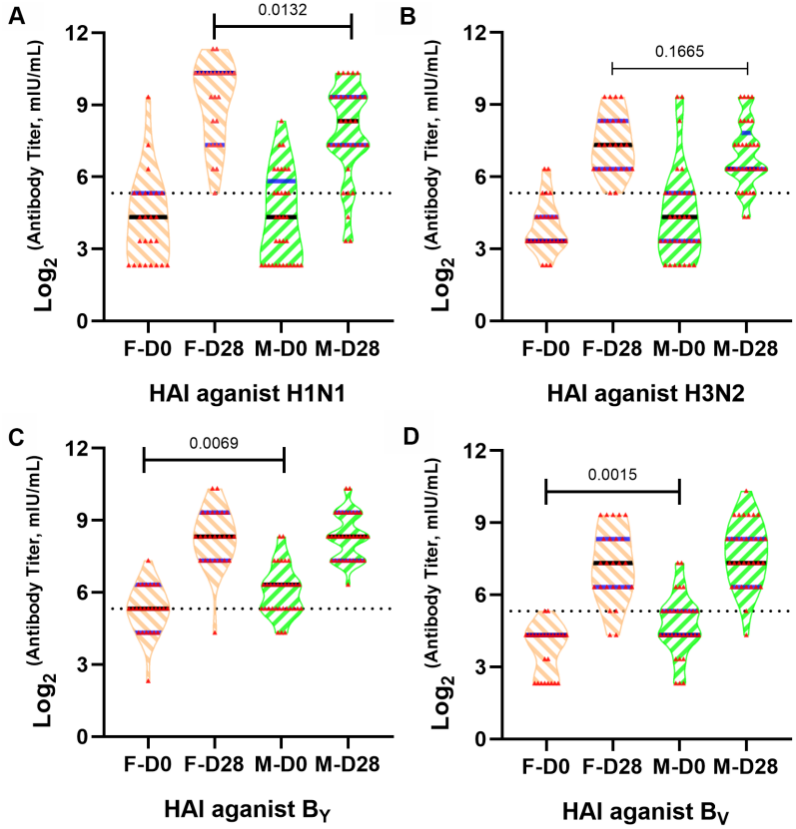
**Different influenza A/H1N1 antibody titers in old males and females after QIVs vaccination.** Violin plots are shown for immune responses of influenza ((**A**) A/H1N1, (**B**) A/H3N2, (**C**) BYAM, (**D**) BVIC)-reactive antibody in subject sera before and after vaccination based on HAI assay, p-value <0.05 indicated statistical significance. The dotted line represents the threshold of protective antibody titer ≥40 against each influenza vaccine strain.

To explore the influences of sex differences on humoral immune response to QIVs, HAI assay was performed to compare humoral immune responses between old females and males. The HAI antibody titers were detected in 58 participants (43.1% female) at 0 and 28 days after inoculation (Figure 1). While male and female subjects had similar healthy state and age (Table 1), we found significant difference (*p*=0.0132) of HAI antibody titer between females and males at 28 days post-vaccination against vaccine H1N1 strain of QIVs formulation (Figure 1A).

**Table 1:** Characteristics of baseline information of subjects.

	**Males**	**Females**	***p*-Value**
**Number of Participants**	33	25	**/**
**Age Range (Median), yrs**	60-80 (67)	61-75 (66)	0.278
**BMI Range (Median)**	17.9-26.2 (22.6)	18.6-33.3 (23.1)	0.261
**Difference Value of Blood Pressure (Median), mmHg**	30-80 (51)	30-77 (50)	0.264

BMI=body mass index.

Females demonstrated higher median antibody titer after inoculation with QIVs against homologous vaccine H1N1 and H3N2 strains, with significant statistical difference (*p*=0.0132) against H1N1 strain at 28 days post-vaccination. The humoral immunity against H1N1 and H3N2 vaccine strains was equivalent between females and males before vaccination. Nevertheless, males had stronger immune responses to B Yamagata lineage wild type virus and B Victoria lineage wild type virus strains compared to the females before vaccination (*p*=0.0069 and *p*=0.0015, respectively) (Figure 1C, 1D), consistent with the investigation that males could sustain higher antibody titer after influenza infection or immunization [18]. No statistical difference of immune response was observed in the different sex groups to the B_YAM_ and B_VIC_ wild type strains after inoculation with the QIVs (*p*>0.05).

### Differential Expression Genes (DEGs) in old adults before and after vaccination

**Figure 2:**
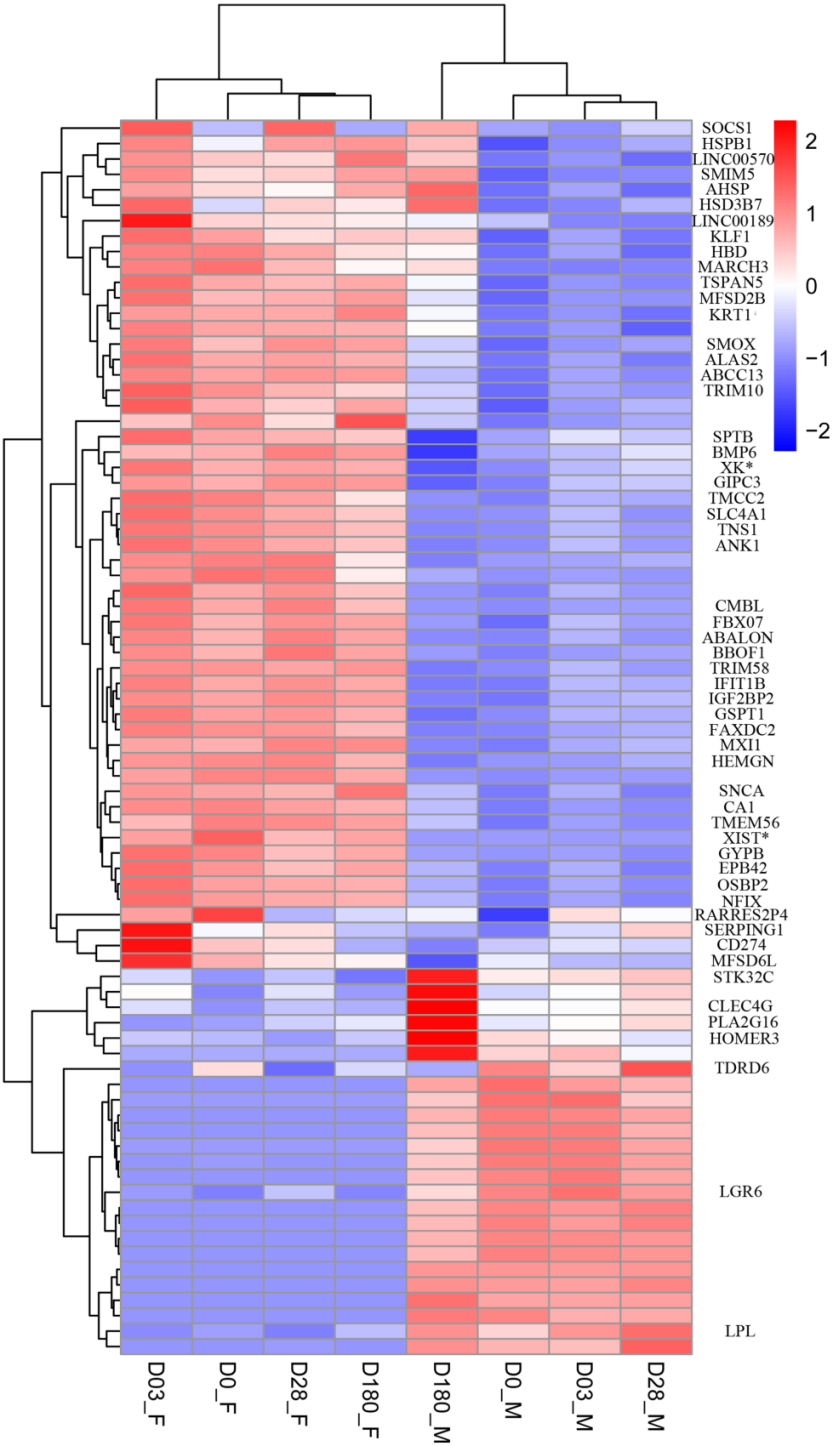
**Heat map of DEGs between female and male group at Day 0 pre-vaccination, Day 03, Day 28 and Day 180 post-vaccination.** The threshold was set with rigorous value for the FDR (false discovery rate, padj) < 0.01 and |log2fold change| > 1. Rows of the gene counts were normalized to set the mean =0, with the standard deviation (SD)=1. The upregulated genes were colored in red, while the downregulated genes were colored in blue.

To investigate sex-based differential transcriptome related to increased adverse reaction in females and lower immune response in males after immunization with QIVs, we analyzed whole blood transcriptome of sixteen older adults using next-generation sequencing. DEGs between two sex groups were distinguished by hierarchical clustering method (Figure 2).

At the Day 0 pre-vaccination, 34 upregulated genes and 17 downregulated genes were identified in female compared to male group. At the Day 3, 20 upregulated genes and 14 downregulated genes were identified in female compared to male group. We found 41 and 25 DEGs in the female group, compared to the male group at Day 28 and Day 180, respectively (Supplementary Figure 1). Among thirteen DEGs identified at four time points, only one gene *FAXDC2* was on the autosome 5, contributing to the biological processes including oxidation-reduction process, lipid biosynthetic process and sterol biosynthetic process (Supplementary Figure 2).

Multiple DEGs were identified at each time point pre- and post-vaccination (listed in Supplementary Figure 1). More genes were significantly upregulated on Day 0, Day 3 and Day 28 in female group compared to the male counterpart, which may result in variant humoral immune responses in sex groups (Figure 2).

Based on the threshold standard, 80 DEGs were filtered for subsequent analysis. Sex-specific gene transcription was minor in the analysis, with 75% of 16 gene detected only in males with FPKM mean values of transcription < 2, consistent with early reports that significantly fewer genes and pathways were affected in males after influenza infection or vaccination [4, 7, 15].

**Figure 3:**
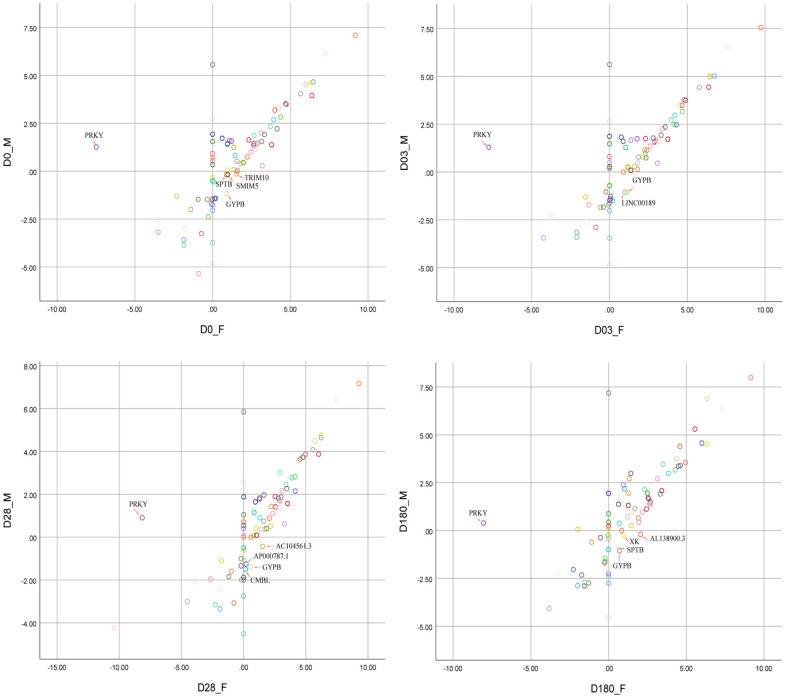
**Scatter plot analysis of 80 DEGs between females and males at four time points (Day 0, Day 03, Day 28, Day 180).** The red arrows pointed to the genes with significant sex-bias. FPKM values were normalized and transformed into Log2|FPKM| for visualization before scatter plot analysis.

The schematic diagram of DEGs with sex-bias was shown in Figure 3. The female-bias *GYPB* gene was highly expressed at four time points, which was activated after immunization and reached the highest expression level at Day 3 (Table 2, females vs. males (Log_2_ |FoldChange|) =2.14). The DEGs with sex-bias, dynamically expressed at four time points, which might mirror the development of female-biased higher HAI titers to influenza A/H1N1 vaccine virus (Table 2).

**Table 2:** List of DEGs that met threshold criteria.

**Gene name**	**Gene descriptions**	**HGNC accession number**	**Log_2_FoldChange (females vs. males) D0/D03/D28/D180**	**Chromosome**	**Gene strand**
ALAS2	5'-aminolevulinate synthase 2	397	2.19/2.19/2.11/1.07	X	-
FBXO7	F-box protein 7	13586	1.11/1.02/1.03/0.77	22	+
AHSP	alpha hemoglobin stabilizing protein	18075	1.49/1.49/1.25/-0.76	16	+
SLC4A1	solute carrier family 4 member 1 (Diego blood group)	11027	1.81/1.71/1.58/1.15	17	-
TRIM58	tripartite motif containing 58	24150	1.70/1.43 /1.45/1.61	1	+
HBD	hemoglobin subunit delta	4829	2.44/1.94/2.16/0.14	11	-
RPS4Y1	ribosomal protein S4 Y-linked 1	10425	0	Y	+
SNCA	synuclein alpha	11138	1.61/1.34/1.49/1.24	4	-
MXI1	MAX interactor 1, dimerization protein	7534	1.25/1.02/1.11/1.18	10	+
GSPT1	G1 to S phase transition 1	4621	1.19/1.14/1.04/1.09	16	-
HSPB1	heat shock protein family B (small) member 1	5246	0.82/1.18/0.90/0.14	7	+
SMOX	spermine oxidase	15862	0.93/1.01/0.87/0.56	20	+
EPB42	erythrocyte membrane protein band 4.2	3381	1.56/1.53/1.31/0.93	15	-
KRT1	keratin 1	6412	1.94/1.83/2.01/0.82	12	-
OSBP2	oxysterol binding protein 2	8504	1.24/1.23/1.11/0.70	22	+
KLF1	Kruppel like factor 1	6345	1.38/1.25/0.94/-0.13	19	-
CA1	carbonic anhydrase 1	1368	2.42/2.03 /1.97/1.09	8	-
IFIT1B	interferon induced protein with tetratricopeptide repeats 1B	23442	1.42/1.24/1.21/1.26	10	+
SERPING1	serpin family G member 1	1228	0.80/1.66/-0.09/0.17	11	+
TSPAN5	tetraspanin 5	17753	1.13/1.16/0.91/0.25	4	-
TMCC2	transmembrane and coiled-coil domain family 2	24239	1.62/1.39/1.14/0.76	1	+
HEMGN	hemogen	17509	1.44/1.35/1.28/1.24	9	-
FAXDC2	fatty acid hydroxylase domain containing 2	1334	1.45/1.32/1.15/1.10	5	-
IGF2BP2	insulin like growth factor 2 mRNA binding protein 2	28867	1.21/1.03/0.98/1.06	3	-
AL356968.2	amyotrophic lateral sclerosis 2 (juvenile) chromosome region, candidate 2 (ALS2CR2) pseudogene	/	1.58/1.55/1.52/1.03	1	+
NFIX	nuclear factor I X	7788	1.29/1.30/1.14/0.61	19	+
AL138900.3	novel transcript	/	2.91/2.61/2.65/2.06	1	+
BMP6	bone morphogenetic protein 6	1073	0.70/0.56/0.63/1.26	6	+
TNS1	tensin 1	11973	1.47/1.25/1.24/1.01	2	-
LINC00570	long intergenic non-protein coding RNA 570	43717	1.52/1.60 /1.46/0.34	2	+
PLA2G16	phospholipase A2 group XVI	17825	-0.48/-0.72/-0.54/-1.51	11	-
SOCS1	suppressor of cytokine signaling 1	19383	0.12/1.10/0.78/-0.66	16	-
ANK1	ankyrin 1	492	1.50/1.27/1.18/1.05	8	-
STK32C	serine/threonine kinase 32C	21332	-0.53/-0.29/-0.49/-1.37	10	-
DDX3Y	DEAD-box helicase 3 Y-linked	2699	0	Y	+
ABALON	apoptotic BCL2L1-antisense long non-coding RNA	49667	1.44/1.37/1.66/1.33	20	+
MFSD2B	major facilitator superfamily domain containing 2B	37207	1.04/1.08/0.871/0.42	2	+
HOMER3	homer scaffold protein 3	17514	-0.44/-0.24/-0.30/-1.18	19	-
LGR6	leucine rich repeat containing G protein-coupled receptor 6	19719	-1.10/-1.07/-0.70/-0.70	1	+
CD274	CD274 molecule	17635	0.61/1.32 /0.40/0.40	9	+
TRIM10	tripartite motif containing 10	10072	1.53/1.35 /1.05/0.31	6	-
AC104561.3	novel transcript	/	1.69/1.66 /1.96/0.48	8	-
PEAR1	platelet endothelial aggregation receptor 1	33631	0.83/0.77/0.76/1.62	1	+
BBOF1	basal body orientation factor 1	19855	1.29/1.31 /1.34/1.12	14	+
EIF1AY	eukaryotic translation initiation factor 1A Y-linked	3252	0	Y	+
SMIM5	small integral membrane protein 5	40030	1.16/1.28/0.95/-0.18	17	+
XK	X-linked Kx blood group	12811	0.91/0.88/0.61/1.04	X	+
SPTB	spectrin beta, erythrocytic	11274	1.06/0.94/0.75/1.49	14	-
KDM5D	lysine demethylase 5D	11115	0	Y	-
GYPB	glycophorin B (MNS blood group)	4703	2.11/2.14/1.94/1.56	4	-
PRKY	protein kinase Y-linked (pseudogene)	9444	-8.75/-9.06/-9.06/-8.43	Y	+
XIST	X inactive specific transcript	12810	†	X	-
LINC00189	long intergenic non-protein coding RNA 189	18461	0.63/2.05/1.12/0.14	21	+
UTY	ubiquitously transcribed tetratricopeptide repeat containing, Y-linked	12638	0	Y	-
AP000787.1	uncharacterized LOC101929295 [Source:NCBI gene;Acc:101929295]	/	1.62/1.32 /1.45/1.13	11	+
MARCH3	membrane associated ring-CH-type finger 3	28728	1.55/1.48/1.13/-0.27	5	-
CMBL	Carboxy methylene buteno lidase homolog	25090	1.61/1.70/1.65/1.13	5	-
TMSB4Y	thymosin beta 4 Y-linked	11882	0	Y	+
TXLNGY	taxilin gamma pseudogene, Y-linked	18473	0	Y	+
GIPC3	GIPC PDZ domain containing family member 3	18183	1.13/0.81 /0.79/1.45	19	+
USP9Y	ubiquitin specific peptidase 9 Y-linked	12633	0	Y	+
ZFY	zinc finger protein Y-linked	12870	0	Y	+
ABCC13	ATP binding cassette subfamily C member 13 (pseudogene)	16022	2.11/1.64/1.81/1.00	21	+
ZP3	zona pellucida glycoprotein 3	13189	-0.51/0.05/0.01/-1.89	7	+
HSD3B7	hydroxy-delta-5-steroid dehydrogenase, 3 beta- and steroid delta-isomerase 7	18324	0.59/1.26/0.61/-0.49	16	+
MFSD6L	major facilitator superfamily domain containing 6 like	26656	0.54/1.44/0.65/1.23	17	-
LINC00278	long intergenic non-protein coding RNA 278	38712	0	Y	+
AC010615.2	uncharacterized LOC105372321 [Source:NCBI gene;Acc:105372321]	/	2.54/2.01 /2.29/1.07	19	-
TTTY15	testis-specific transcript, Y-linked 15	18567	0	Y	+
RARRES2P4	retinoic acid receptor responder 2 pseudogene 4	48703	4.45/0.38/-0.69/-0.59	4	-
AL157756.1	uncharacterized LOC101927702 [Source:NCBI gene;Acc:101927702]	/	1.25/1.14/0.57/0.45	14	-
AC114811.2	novel transcript, antisense TSPAN5	/	2.03/1.30/0.88/1.06	4	+
TMEM56	transmembrane protein 56	26477	1.73/1.05/1.44/0.76	1	+
AC010889.1	novel transcript	/	0	Y	-
LPL	lipoprotein lipase	6677	-0.95/-1.49/-1.80/-0.98	8	+
ANOS2P	anosmin 2, pseudogene	6214	0	Y	+
BCORP1	BCL6 corepressor pseudogene 1	23953	0	Y	-
TDRD6	tudor domain containing 6	21339	-0.32/-0.81/-1.526/0.14	6	+
TTTY14	testis-specific transcript, Y-linked 14	18495	0	Y	-
NLGN4Y	neuroligin 4 Y-linked	15529	0	Y	+

Note: The values of Log2|FoldChange| column including expression level were compared between females and males at four different time points (Day 0, Day 03, Day 28, Day 180), ‘0' represented the transcripts only detected in males on Y chromosome, and '†' indicated the transcript only detected in females on X chromosome.

Based on gene clustering and differential analysis, 19, 14, 17 and 12 genes showed differential expression over ten-fold changes between two sex groups at Day 0 pre-vaccination, Day 3, Day 28, as well as Day 180 post-vaccination, respectively. The majority of those genes were upregulated in male group, which are distributed on the Y chromosome (Table 2). The gene transcription intensity detected as sexual dimorphism varied at each time point, with moderate fold-changes detected on the autosomes, and wide variant fold-changes detected on X chromosome.

DEGs with sex-bias over two-fold change and *p*adj <0.05 were analyzed by various databases including Kyoto Encyclopedia of Genes and Genomes (KEGG), DisGeNET, Reactome, Disease Ontology (DO), and Gene Ontology (GO) to investigate biological pathways involved in disease susceptibility and physiological distribution. No significant gene enrichment to the functional pathway was identified by mapping to KEGG database. Analysis based on DisGeNET showed that DEGs upregulated in females were primarily associated with anemia related diseases such as Hemoglobinopathies, Reticulocytosis, Increased bilirubin level, Hereditary spherocytosis and Anemia Hemolytic. DEGs upregulated in males were associated with reduced fertility such as azoospermia and Non-obstructive azoospermia. REACTOME analysis showed two genes *KDM5D* and *UTY* on the Y chromosome related to HDMs demethylate histones had lower expression in male group from Day 0 to Day 180 post-vaccination. DEGs upregulated in females included *TRIM58*, *TRIM10*, *KLF1*, *ALAS2* and *EPB42*, which are mainly associated with erythrocyte differentiation, erythrocyte homeostasis, myeloid cell homeostasis and homeostasis of number of cells, while the genes highly expressed in males as *KDM5D* and *UTY* were enriched to histone lysine demethylation and protein dealkylation pathways.

Analyzing the correlation of these eighty DEGs collected by sex pairing comparison from four time points with female antibody against influenza A/H1N1 vaccine virus, to identify the hub genes contributing to the female-bias humoral immunity. No DEGs was identified significant statistical correlation with female-biased HAI titers to influenza A/H1N1 vaccine virus.

The robustness of RNA-Seq data was validated by the quantitative real-time PCR (qRT-PCR), detecting the relative expression level of interested genes. The RNA-Seq result validation was conducted referring to previous study, and the gene expression patterns of *CD274* and *SOCS1* genes were shown to be consistent with the transcriptome results higher expression in female group (Supplementary Figure 3).

### DEGs associated with innate immune responses to QIVs vaccination in old adults

To further exploit the differences of DEGs patterns between older sex groups, we compared Day 3 transcripts with the baseline Day 0 in females and males, respectively. There were 16 activated and 24 inhibited genes expressed in females in the early stage after immunization, while no genes with significantly different expression were identified in males (Table 3).

**Table 3:** Number of DEGs following QIVs vaccination in females and males.

**Variations of gene transcripts**	**Number of genes**					
	**Innate immunity (D03 vs. D0)**		**adaptive immunity (D28 vs. D0)**		**Antibody attenuation (D180 vs. D0)**	
	**Females**	**Males**	**Females**	**Males**	**Females**	**Males**
**Upregulated**	16	0	6	4	69	1473
**Downregulated**	24	0	202	97	161	352
**All**	40	0	208	101	230	1825

GO enrichment analysis showed that compared with the males, upregulated genes in females were involved in innate immune process, such as type I interferon signaling pathway (*IFI35/USP18/IFITM3/IFI6*, GO:0060337), complement activation of classical pathway (*SERPING1/C1QA/C2*, GO:0006958), but down-regulated genes were not enriched in any biological function. By KEGG analysis, the activated genes (*SERPING1/C1QA/C2*) involved in biological process of complement and coagulation cascades (hsa04610, *p*adj =0.0001), which may contribute to early vaccination responses such as muscle contraction, chemotaxis, phagocyte recruitment and inflammation (Supplementary Figure 4; Complement and Coagulation Cascades, hsa04610).

Hub genes *IFITM3* (co*r*= 0.563, *p*= 0.023) and *C1QA* (co*r*= 0.665, *p*= 0.005) were positive correlated with female-biased HAI titers to influenza A/H1N1 vaccine virus, would play critical role in the innate immune responses to QIVs.

### DEGs associated with adaptive immune responses to QIVs vaccination in old adults

**Figure 4:**
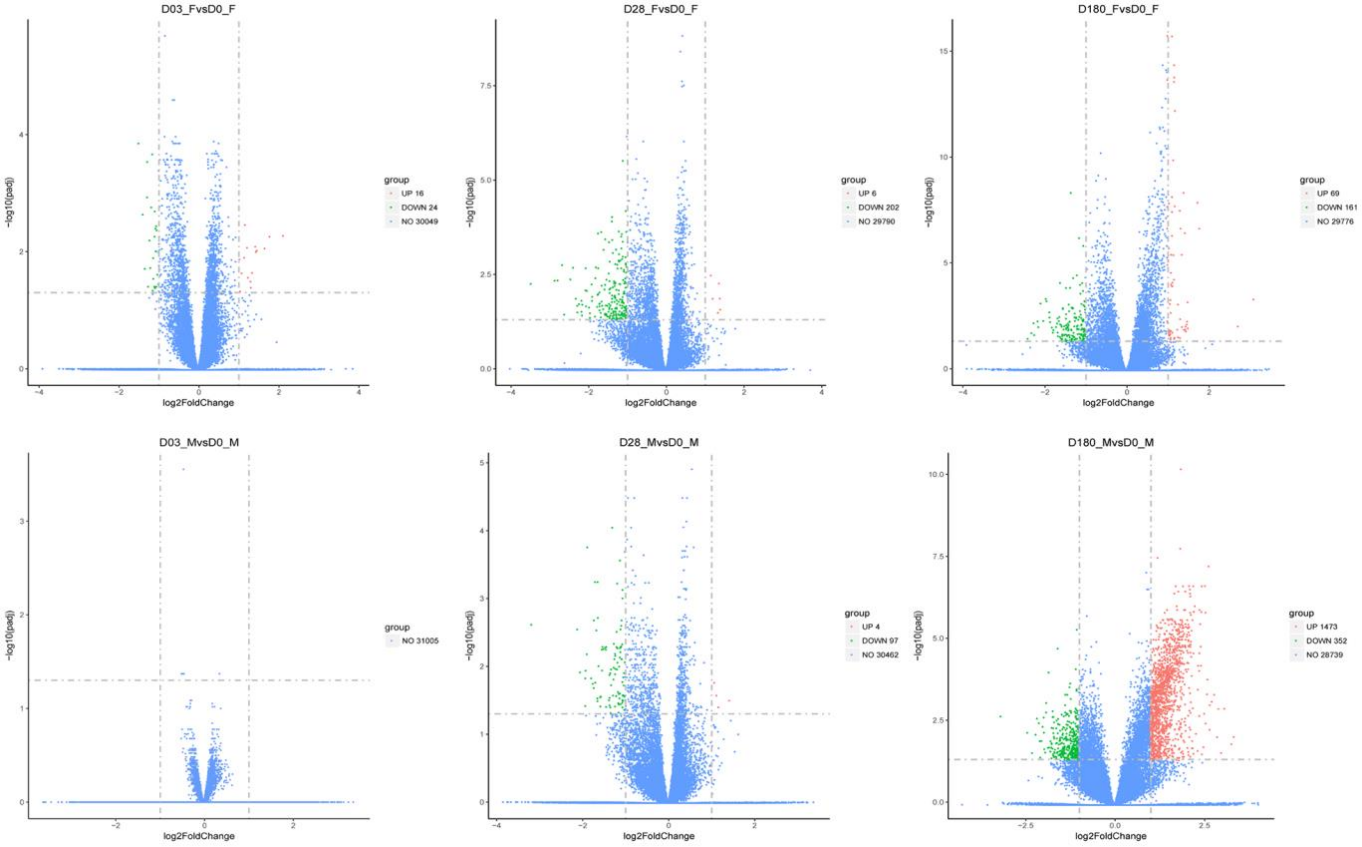
**The volcano plots of DGEs in females and males compared with the baseline (Day 3, Day 28, and Day 180).** The red dots represented upregulated genes, and the blue dots represented genes with no significant change, while the green dots represented downregulated genes. The threshold was set with rigorous value for the FDR (false discovery rate, padj) < 0.05 and |log_2_fold change| > 1. The x-axis denoted the fold changes of gene expression relative to baseline, shown with log_2_|fold change|. The y-axis indicated the significance of DEGs.

DEGs associated with adaptive immunity to QIVs vaccination were mostly downregulated both in females and males, at Day 28 post-vaccination compared to the baseline Day 0. Total 208 and 101 DEGs were identified in females and males at Day 28, respectively (Figure 4), with 27 downregulated DEGs shared by females and males. Only 6 genes in females including *IGKV1-12*, *AP001000.1*, *TIPARP-AS1*, *HSPD1P1*, *IGHV2-26* and *AC008429.2* and 4 genes in males including *TRIM36*, *BIRC5*, *C2* and *C1QB* showed higher expression compared to baseline Day 0. The highly expressed genes (*BIRC5/C2/C1QB*) involved in the regulation of protein processing (GO:0070613, *p*adj=3.66E-05) and complement activation of classical pathway (GO:0006958, *p*adj= 0.0004), and enriched in Complement and coagulation cascades (*C2/C1QB*, hsa04610, *p*adj=0.017) KEGG pathway term were confirmed in males.

In females, G-protein coupled peptide receptor activity (GO: 0008528) and peptide receptor activity (GO:0001653) associated genes (*LTB4R2/RXFP4/MC1R/GPR75*) were downregulated at Day 28 compared to the baseline Day 0. Notch receptor 3 (NOTCH3) which contributes to the activation of Th1 cellular immunity, and linker for activation of T cells (LAT) and Fos proto-oncogene (FOS) which directly activate Th2 humoral immunity were significantly repressed in females at Day 28. In males, two genes *AOC3* and *AOC2* were downregulated, associated with molecular function involved in oxidoreductase activity (acting on the CH-NH2 group of donors, oxygen as acceptor, GO:0016641), which impacted biological process of cellular response to xenobiotic stimulus (GO:0071466).

Hub gene *IGHV2-26* (co*r*= 0.524, *p*= 0.037) was positive correlated with female-biased HAI titers to influenza A/H1N1 vaccine virus, would play critical role in the adaptive immune responses to QIVs.

### Variability of DEGs and biological function pathways associated with decline of humoral antibody titers to QIVs vaccination

Previous studies reported that sustained capacity of protective antibody titers specific to influenza vaccine strains was poor in older adults [19, 20]. In females, GO enrichment analysis for DEGs at Day 180 compared to baseline Day 0 showed that primary DEGs were involved in the process associated with endoplasmic reticulum activity, including protein targeting to ER (GO:0045047), rRNA processing (GO:0006364), polysomal ribosome (GO:0042788) and cytoplasmic side of endoplasmic reticulum membrane (GO:0098554) (Table 4). Differential analysis at Day 180 compared with Day 28 in females showed that DEGs were enriched to multiple terms related to regulation of leukocyte activity including the biological process of positive regulation of myeloid leukocyte differentiation (GO:0002763) and Fc-epsilon receptor signaling pathway (GO:0038095) during the decline stage of specific antibody (Table 4). On the other hand, DEGs in males at Day 180 compared to baseline Day 0 were enriched for GO terms including protein targeting to membrane (GO:0006612), oxidative phosphorylation (GO:0006119), cellular protein complex disassembly (GO:0043624) and protein degradation (Table 4).

**Table 4:** Significantly enriched immune relevant GO terms in old females and males at Day 180.

**Contrast**	**Sex**	**GO ID**	**Description**	**Category**	**Genes^‡^**	***p*adj**
D180 vs. D0	Female	GO:0045047	protein targeting to ER	BP	14	5.77E-18
		GO:0006364	rRNA processing	BP	16	1.19E-14
		GO:0042788	polysomal ribosome	CC	6	5.31E-08
		GO:0098554	cytoplasmic side of endoplasmic reticulum membrane	CC	4	3.08E-06
		GO:0005924	cell-substrate adherent junction	CC	6	0.049794522
		GO:0003735	structural constituent of ribosome	MF	14	3.19E-13
	Male	GO:0006612	protein targeting to membrane	BP	82	7.94E-39
		GO:0006119	oxidative phosphorylation	BP	58	9.48E-27
		GO:0043624	cellular protein complex disassembly	BP	54	4.69E-13
		GO:0010257	NADH dehydrogenase complex assembly	BP	27	1.93E-11
		GO:0032981	mitochondrial respiratory chain complex I assembly	BP	27	1.93E-11
		GO:0031146	SCF-dependent proteasomal ubiquitin-dependent protein catabolic process	BP	22	2.36E-06
		GO:0006521	regulation of cellular amino acid metabolic process	BP	17	7.53E-05
		GO:0010499	proteasomal ubiquitin-independent protein catabolic process	BP	9	0.002674681
		GO:1903321	negative regulation of protein modification by small protein conjugation or removal	BP	17	0.002681796
		GO:0006959	humoral immune response	BP	29	0.005762827
		GO:0042590	antigen processing and presentation of exogenous peptide antigen via MHC class I	BP	17	0.006188976
		GO:0045116	protein neddylation	BP	6	0.031860865
		GO:0050852	T cell receptor signaling pathway	BP	29	0.036706782
		GO:2001242	regulation of intrinsic apoptotic signaling pathway	BP	24	0.043430667
		GO:0000502	proteasome complex	CC	18	5.02E-05
D180 vs. D28	Female	GO:0002763	positive regulation of myeloid leukocyte differentiation	BP	1	0.017610107
		GO:0038095	Fc-epsilon receptor signaling pathway	BP	1	0.020494521
		GO:0001047	core promoter binding	MF	1	0.016815814
	Male	GO:0045047	protein targeting to ER	BP	65	5.57E-55
		GO:0006119	oxidative phosphorylation	BP	49	5.97E-28
		GO:0031146	SCF-dependent proteasomal ubiquitin-dependent protein catabolic process	BP	13	0.003315273
		GO:0031640	killing of cells of other organism	BP	9	0.006461521
		GO:2001244	positive regulation of intrinsic apoptotic signaling pathway	BP	10	0.01646663
		GO:1902750	negative regulation of cell cycle G2/M phase transition	BP	14	0.032386761
		GO:0097526	spliceosomal tri-snRNP complex	CC	9	0.00013242
		GO:0005839	proteasome core complex	CC	5	0.030643822
		GO:0003735	structural constituent of ribosome	MF	94	1.90E-78
		GO:0140104	molecular carrier activity	MF	8	0.020074571
		GO:0004298	threonine-type endopeptidase activity	MF	5	0.048464518

Note: ‘‡’ indicated the number of genes enriched in the GO terms.

KEGG pathway enrichment analysis in males at Day 180 compared to baseline Day 0 showed that DEGs were involved in regulation of cell cycle, cell differentiation and signal transduction pathways including oxidative phosphorylation, thermogenesis, proteasome and pyrimidine metabolism pathways, but no significant enriched immune response pathway was identified in the females.

## DISCUSSION

**Figure 5:**
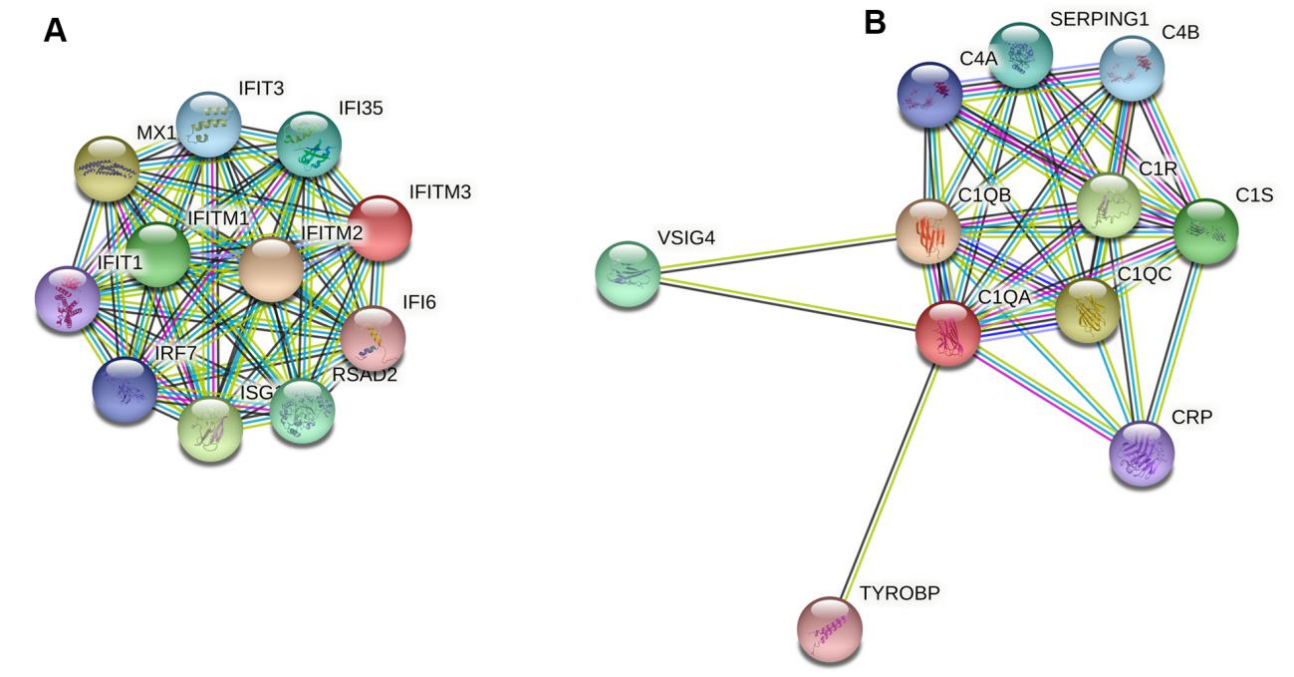
Protein-protein interaction (PPI) network analysis of two female-bias hub genes *IFITM3* (**A**) and *C1QA* (**B**). The hub gene *IFITM3* was involved in type I interferon signaling pathway, while gene *C1QA* contributed to complement activation of classical pathway.

In this study we found that DEGs were associated with immune responses to QIVs vaccination, consistent with previous studies that sex dimorphism would impact immune responses to vaccines in older people [14, 21]. The immune responses to vaccination initiated innate immunity earlier and stronger in females than males, which may enhance vaccine efficacy in older females. At Day 3 post-vaccination compared with the baseline Day 0, GO terms enriched for DEGs in females were primarily relevant to type I interferon signaling, complement activation and regulation of acute inflammatory response, revealing early features of immune responses in older females. The efficiency of innate immunity in the aged was delayed, and we identified 10 hub genes (*FCGR1A/ANKRD22/BATF2/SERPING1/IFI35/IFI6/IFITM3/C1QA/C2/USP18*), which mainly attributed to immune pathways. Two of 10 critical genes (*IFITM3* and *C1QA*) identified positive correlation with female-bias HAI titers to influenza A/H1N1 vaccine virus, genes interacted with these two hub genes were displayed (Figure 5). The complement activity was also noted in the upper respiratory tract infection with influenza [22].

Compared with the baseline Day 0, on Day 28 almost all DEGs were downregulated, except 6 and 4 genes were upregulated in females and males, respectively. The 6 upregulated genes in females included 2 of 3 pseudogenes *AP001000.1* and *HSPD1P1* belonged to heat shock protein family, 2 IG-V genes (immunoglobulin variable domain gene) *IGKV1-12* and *IGHV2-26* that may contribute to extensive antibody diversity and account for higher antibody titer in females than males [23], and one gene *TIPARP-AS1* is a long non-coding RNA with unknown function [24]. For the 4 genes upregulated in males, androgen-influenced *TRIM36* could regulate the activity of *MAPK/ERK/MSK-1* pathway [25], and the other three genes (*BIRC5/C2/C1QB*) could participate in classical complement activation pathway, although the inhibition of the complement activity would alleviate acute H5N1 influenza infected lung injury [26], consistent with higher morbidity and mortality in immunosenescent males.

After enrichment analysis of downregulated genes in older females, four genes (*LTB4R2/RXFP4/MC1R/GPR75*) were significantly enriched in biological process involved in G-protein coupled peptide receptor activity and peptide receptor activity, and three transcripts (NOTCH3/LAT/FOS) were associated with Th1 and Th2 cell differentiation. In fact, lower expression of FOS could contribute to a higher incidence due to influenza virus in the elderly [27].

A shared profile between older females and males was the initiation of biological process involved in classical complement activation pathway (GO:0006958) post-vaccination, although immune response was delayed in males compared to females. Complement C3 protein played a protective role in host defense to pandemic 2009 H1N1 and H5N1 influenza A virus infection, while the opposite effects of complement component C5 in mice may contribute to severe influenza H1N1 infections [28]. These studies demonstrated that complement is an important host defense mechanism that play a critical role in influenza virus neuralization [29].

Profiling of differential gene expression facilitated the understanding of differential immune responses to QIVs vaccination between older females and males at different time points. The first significant sex dimorphic difference was that females showed faster and stronger immune responses than males. The second discrepancy was that a certain number of sex-bias genes were highly expressed only in one sex. The early upregulated genes in older females were enriched in GO and KEGG terms and associated with innate and acquired immune responses, consistent with the terms enriched for the activated genes in older males. The highly expressed genes were vital to initiate the expression and release of inflammatory cytokines or the host innate immunity, thus females may potentially have more sensitive and stronger immune responses in the early days of vaccination with QIVs than males. The downregulated genes (*PLIN4/FABP3*) in females were related to PPAR signaling pathway, which may impact the vaccine efficacy. These results showed that genes associated with innate immunity had higher transcription whereas genes relevant to cellular senescence had lower transcription in females than males post-vaccination. We found that most DEGs were repressed on Day 28 in both females and males, and more upregulated genes were identified in males at Day 180 than females. These features suggest that males have the advantages to sustain a durable response to QIVs vaccination, while older females have a rapid decline of antibody levels.

A few studies focused on sex disparities of humoral immunity with vaccines. Fink et al. [21] proposed that the guidelines such as formulations and dosage for vaccines in the elderly should consider sex differences. Engler et al. [30] reported that significantly higher immune responses to influenza vaccines in females than males were identified for all ages, regardless of dose or influenza strain. The stronger immune responses during influenza infection in females resulted in immune mediated pathologies, such as the overexpression of cytokines that contribute to severe lung failure [31]. Females are susceptible to autoimmune diseases, such as systemic lupus erythematosus. The sexual differences of efficient vaccination in females and males are unclear, and the mechanisms underlying robust immune response to vaccination in males and quick decline of protective antibody in females should be further investigated.

From the perspective of seasonal influenza infection, there are significant differences in morbidity and mortality between females and males [32]. Influenza infection in real world is influenced by multi factors, including influenza vaccination coverage that attributed to vaccination policy, vaccination history, knowledge and attitude toward influenza and vaccination [33], as well as populations of different sex and age. Therefore, transcriptome analysis of the mechanisms underlying sex immune differences is affected by confounding factors. Libert et al. [34] found that X chromosome contains many genes involved in immune function, such as Toll-like receptor 7 (*TLR7*), CD40 ligand (*CD40L*) and forkhead box P3 (*FOXP3*), and loss-of-function mutations on the chromosome would result in impaired immune responses. Genes on Y chromosome plays important role in regulating susceptibility to infectious disease [35]. Further studies should focus on how DEGs influence vaccination efficacy and incidence of influenza infections. The sex-based differences should be taken into consideration to develop safer and more effective seasonal influenza vaccines.

In summary, our study identified that DEGs associated with immune responses involved in type I interferon signaling pathway and classical complement activation pathway had higher and earlier expression at Day 3 in females than in males. These results suggest that females have a greater immune response to QIVs. Furthermore, old males have the advantages to sustain a durable response to influenza vaccination, while old females have rapid decline of antibody levels.

## MATERIALS AND METHODS

### Ethics statement

Before the activity of this clinical study was employed, the written informed consent was obtained from each subject. The study was conducted in accordance with the Declaration of Helsinki, the clinical protocols were approved by the Medical Ethics Committee of Guangdong Centers for Disease Control and Prevention, and conducted according to the local institutional ethics committee guidelines. The trial was registered with China Drug Trials.org.cn (Registration Numbers: CTR20190846; subjects over 60 yrs). This study population and laboratory detection protocols were described previously [36–39].

### Clinical study organization

The initial study cohort recruited 60 physically healthy older adults of Han Chinese (median age 67 years and age range 60-80 years, 43.3% female) who were inoculated by intramuscular injection at nondominant arm with the 2018-2019 seasonal QIVs (Wuhan Institute of Biological Products Co., Ltd., lot SH201805649) or control vaccine (Hualan Biological Engineering Co., Ltd., lot 201809B033; containing the A/Michigan/45/2015 NYMC X-275A [H1N1; an A/Michigan/45/2015(H1N1)pdm09 like virus], A/Singapore/INFIMH-16-0019/2016 IVR-186 [H3N2; an A/Singapore/INFIMH-16-0019/2016(H3N2) like virus], B/Phuket/3073/2013 wild type virus [B Yamagata lineage], and B/Colorado/06/2017 wild type virus [B Victoria lineage]). Whole blood samples (approximate 4 ml each) of the subjects were promptly collected at Day 0 (pre-vaccination), 3-, 28- and 180-days post-vaccination with EDTA-K2 contained tubes. Hemagglutination-inhibiting (HAI) antibody titers were detected from 58 participants (43.1% female) at 0 and 28 days post-inoculation, and physical examination records of all subjects were collected. Subsequently, 16 of 58 subjects were selected for transcriptome analysis of whole blood samples, and all the data were applied to statistical correlation analysis. The characteristics of subjects enrolled was listed in Table 1.

### HAI assay

HAI assay was performed in accordance with standard WHO procedures [40]. A 1% suspension of chicken erythrocytes and 4 HA units/25 μl of corresponding influenza virus antigens were employed in the detection of functional antibody titers. Serum samples were treated with receptor destroying enzyme and tested in duplicate in serial 2-fold dilutions initiating from 1:10. Each plate contained both negative and positive serum controls. The HI titer was defined as the inverse of the highest serum dilution to inhibit hemagglutination. For the convenience of statistical analysis, HI antibody titers below 10 were treated as 5.

### RNA isolation and mRNA sequencing

Total RNA was extracted from whole blood samples using TRIzol (Bio Basic Inc.) according to the manufacturer’s instructions, and analyzed using RNA Nano 6000 Assay Kit of the Bioanalyzer 2100 system (Agilent Technologies, CA, USA) and Qubit® RNA Assay Kit in Qubit® 2.0 Flurometer (Life Technologies, CA, USA). mRNA sequencing libraries were built using 3 μg RNA and NEBNext® UltraTM RNA Library Prep Kit for Illumina® (NEB, USA) following the manufacturer’s recommendations. Poly-T oligo-attached magnetic beads were employed to isolate poly-A RNA. First strand cDNA was synthesized using random hexamer primer and M-MuLV Reverse Transcriptase (RNase H-). Second strand cDNA synthesis was subsequently performed using DNA Polymerase I and RNase H. After adenylation of 3’ends of DNA fragments, NEBNext Adaptor with hairpin loop structure were ligated to prepare for hybridization. At last, PCR products were purified (AMPure XP system) and library quality was assessed on the Agilent Bioanalyzer 2100 system. The clustering of the index-coded samples was performed on a cBotCluster Generation System using TruSeq PE Cluster Kit v3-cBot-HS (Illumia) according to the manufacturer’s instructions. After cluster generation, the library preparations were sequenced on an Illumina Hiseq platform (Illumina NovaSeq 6000) and 125 bp/150 bp paired-end reads were generated.

Hisat2 v2.0.5 was used for building index of the GRCh38 (hg19) reference genome, as well as the alignment of paired-end clean reads to the reference genome. The Conditional Quantile Normalization was applied to gene counts to adjust for GC content and gene length. Genes numbers mapped to each gene were counted by FeatureCounts v1.5.0-p3. Subsequent analyses were based on the normalized values.

### Differential expression analysis

(For DESeq2 with biological replicates) Differential expression analysis of two conditions/groups (two biological replicates per condition) was performed using the DESeq2 R package (1.16.1). The *P*-values were adjusted using the Benjaminiand Hochberg’s approach for controlling false rate. Genes with an adjusted *P*-value <0.05 found by DESeq2 were assigned as differentially expressed.

### GO and KEGG enrichment analysis of differentially expressed genes

Gene Ontology (GO) enrichment analysis of differentially expressed genes was implemented by the Cluster Profiler R package, in which gene length bias was corrected. GO terms with corrected *P*-value < 0.05 were considered significantly enriched by differential expressed genes. Cluster Profiler R package was used to test the statistical enrichment of differential expression genes in KEGG pathways (http://www.genome.jp/kegg/).

### Protein-Protein Interactions (PPI) analysis of differentially expressed genes

PPI analysis of differentially expressed genes was based on the STRING database to predict Protein-Protein Interactions. Cytoscape version 3.7.2 software was used for gene network visualization.

### Data availability statement

RNA-Seq Expression data in our study were deposited at the National Center for Biotechnology Information Gene Expression Omnibus (GEO) public repository (accession number: GSE151558), to review GEO accession GSE151558: https://www.ncbi.nlm.nih.gov/geo/query/acc.cgi?acc=GSE151558.

### Statistical analysis

Statistical analyses were conducted using IBM SPSS Statistics 26.0.0.0 (SPSS Software). Spearman’s correlation analysis was performed, and p values < 0.05 were considered significant. Independent-sample t test was employed to compare two groups, with a 95% confidence interval. A least significant difference (LSD) of the post-hoc test of one-way analysis of variance (ANOVA) was used to compare multiple groups at a confidence interval of 95%.

## Supplementary Material

Supplementary Figures
