# Supplementary material for: Sex-specific differences of humoral immunity and transcriptome diversification in older adults vaccinated with inactivated quadrivalent influenza vaccines
Source: Aging (Albany NY). 2021 Mar 19;13(7):9801–19. doi: 10.18632/aging.202733 (PMC8064175; doi:10.18632/aging.202733)
Supplement: Supplementary Figures [file aging-13-202733-s001.pdf]

SUPPLEMENTARY FIGURES

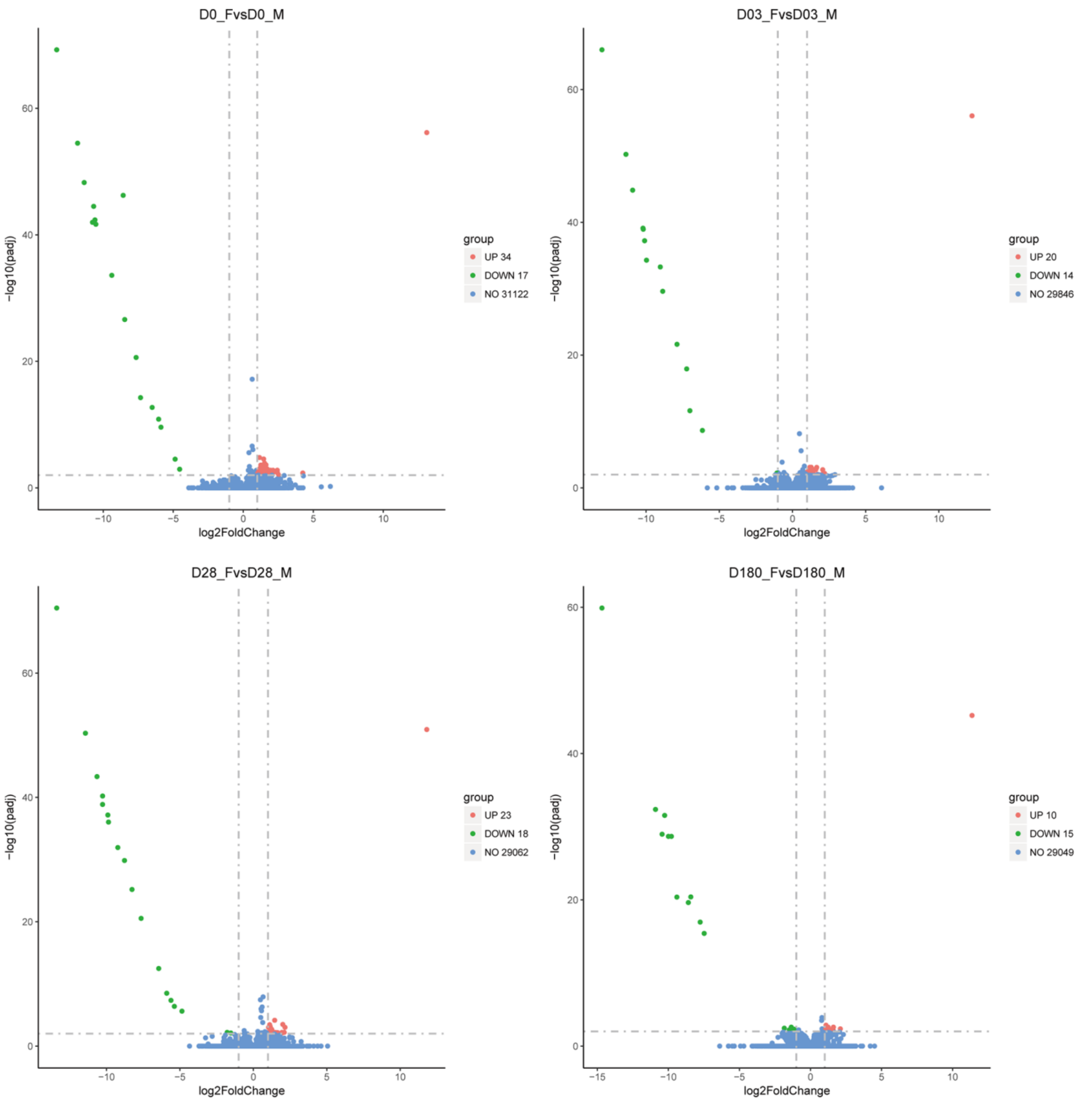

Supplementary Figure 1. The volcano plots of the differentially expressed genes between females and males at four time points (Day 0, Day 3, Day 28, and Day 180). The red dots represent the up-regulated genes, and the blue dots show the no significant-change genes, while the genes with significantly altered down-regulated expression are annotated as green. The threshold was set with rigorous value for the FDR (false discovery rate, padj) < 0.01 and |log2 fold change| > 1. The x-axis denoted the fold changes of gene expression relative to baseline, showed with log2|fold change|, and the y-axis indicated the significance of the differential expressed genes.

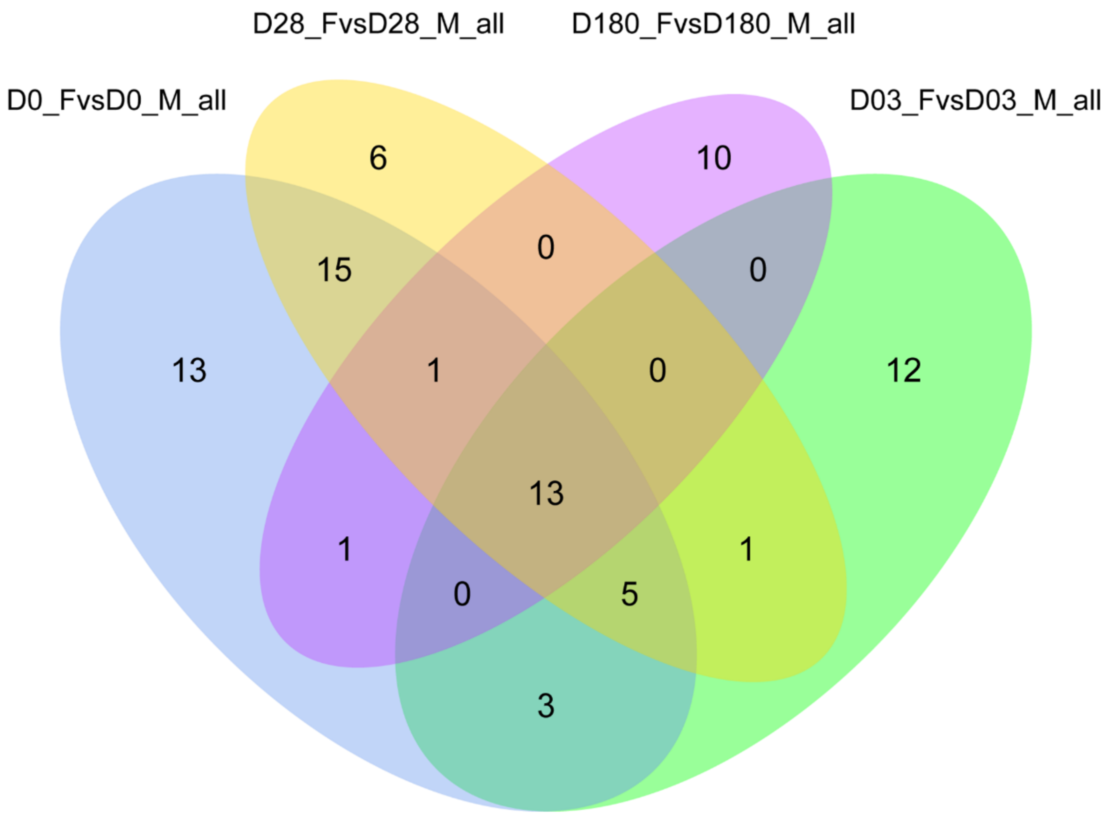

Supplementary Figure 2. The Venn diagram of the differentially expressed genes between females and males at four time points (Day 0, Day 3, Day 28, and Day 180).

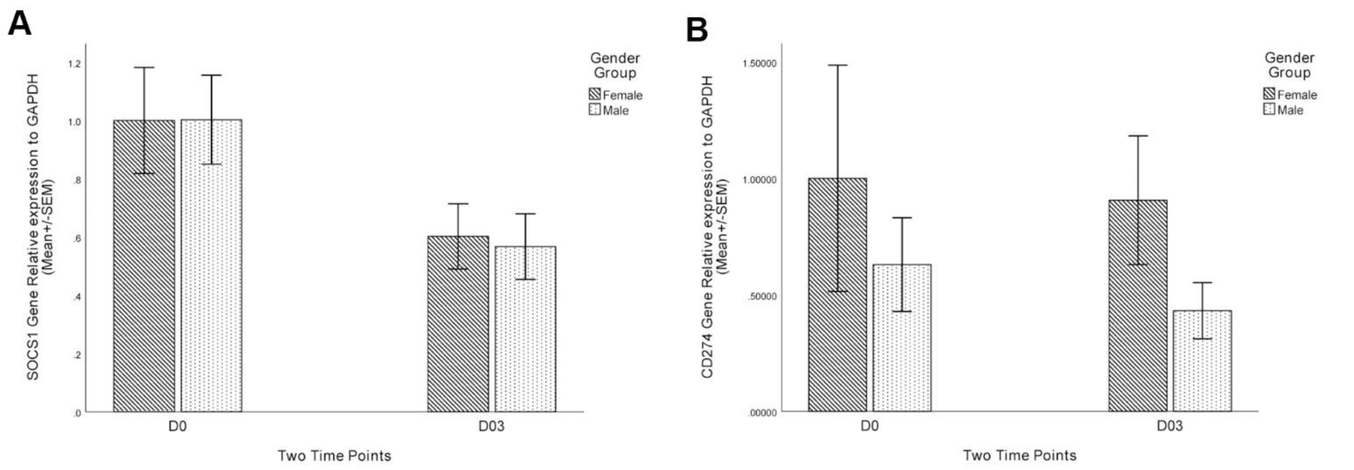

Supplementary Figure 3. Relative expression level of *SOCS1* (**A**) and *CD274* (**B**) genes between sex groups. The expression level of *GAPDH* was employed as an internal control. Each sample was detected in triplicate. Each bar and error bar represent Mean FC (fold-change) value and the Standard Error (SEM), respectively. Statistical significance was considered as *p*-value <0.05.

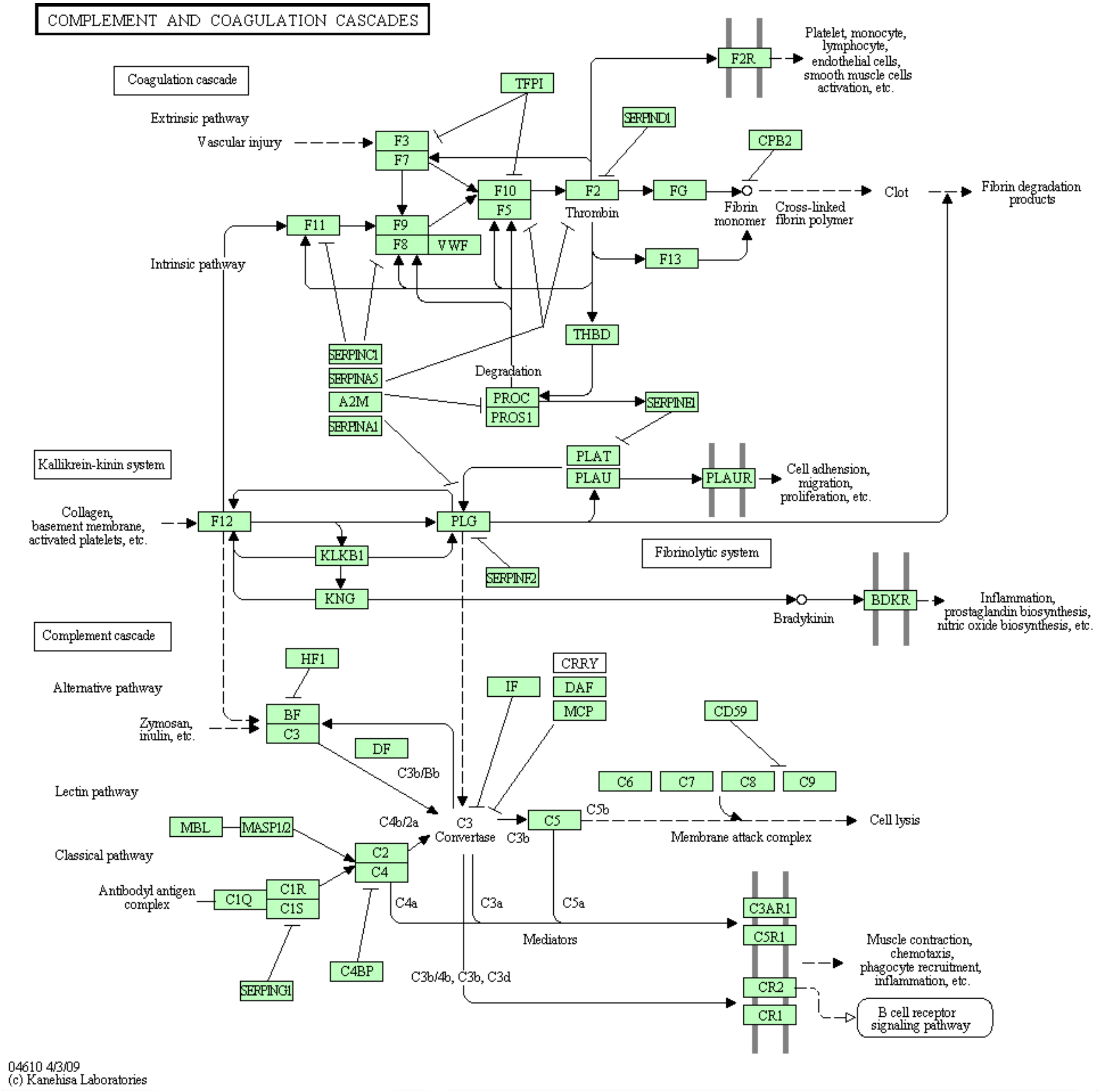

Supplementary Figure 4. Complement and coagulation cascades, hsa04610.
